# The Influence of Anesthetics on the Functions of the Endothelium and Oxidative Stress: A Critical Review

**DOI:** 10.3390/biomedicines13102357

**Published:** 2025-09-26

**Authors:** Marko Djuric, Irina Nenadic, Nina Radisavljevic, Dusan Todorovic, Maja Stojanovic, Nemanja Dimic, Marina Bobos, Suzana Bojic, Predrag Stevanovic, Predrag Savic, Dejan Stojakov, Ivan Palibrk, Dragan Djuric

**Affiliations:** 1 Clinic for Anesthesiology and Intensive Care, University Clinical Hospital Center “Dr Dragisa Misovic-Dedinje”, 11030 Belgrade, Serbia; nemanjadimic@live.com (N.D.); marinamorisan@gmail.com (M.B.); subojic@yahoo.com (S.B.); predrag.stevanovic@med.bg.ac.rs (P.S.); 2 Department of Anesthesiology, Reanimatology and Intensive Care, Faculty of Medicine, University of Belgrade, 11000 Belgrade, Serbia; nenadicirina@gmail.com (I.N.); ivanpalibrk@yahoo.com (I.P.); 3 Institute of Medical Physiology “Richard Burian”, Faculty of Medicine, University of Belgrade, 11000 Belgrade, Serbia; nina_radisavljevic@outlook.com (N.R.); t.dusan@hotmail.com (D.T.); dr_djuric@yahoo.com (D.D.); 4 Dedinje Cardiovascular Institute, 11000 Belgrade, Serbia; majastojanovic05@gmail.com; 5 Clinic for Surgery, University Clinical Hospital Center “Dr Dragisa Misovic-Dedinje”, 11030 Belgrade, Serbia; predrag.savic@dragisamisovic.bg.ac.rs (P.S.); dejan.stojakov@hotmail.com (D.S.); 6 Department of Surgery, Faculty of Medicine, University of Belgrade, 11000 Belgrade, Serbia; 7 Department of Anesthesia and ICU, Clinic for Digestive Surgery, University Clinical Center of Serbia, 11000 Belgrade, Serbia

**Keywords:** anesthetics, cardiovascular diseases, comorbidities, endothelium

## Abstract

Endothelial dysfunction (characterized by reduced vasodilation or vasoconstriction, oxidative stress, inflammation, and pro-thrombotic condition) is a critical factor in the pathophysiology of various cardiovascular conditions, and the application of anesthetics can affect this dysfunction. Patients undergoing major surgery, especially cardiovascular surgery, are at increased risk of endothelial dysfunction. The impact of anesthetics on endothelial function can vary depending on the specific agent, dosage, duration of exposure, comorbidities, etc. Certain anesthetics, especially at higher doses, may increase the production of reactive oxygen species (ROS), leading to oxidative stress and endothelial dysfunction through reduced nitric oxid (NO) availability. Some anesthetics can modulate inflammatory responses, either by suppressing or exacerbating inflammation, or may affect the permeability of the endothelium, potentially leading to pulmonary edema and disruption of the blood-brain barrier. Anesthetics can influence endothelial glycocalyx. Understanding anesthetics effects is crucial for optimizing anesthetic management, particularly in patients with pre-existing cardiovascular issues. Therefore, the aim of this review is to critically evaluate the effects of different classes of anesthetics on endothelial function and oxidative stress. Specifically, we address how anesthetics influence NO bioavailability, endothelial glycocalyx integrity, inflammatory and oxidative pathways, and clinical outcomes in surgical patients. By summarizing current evidence, we aim to highlight mechanistic insights and identify potential perioperative strategies to minimize endothelial dysfunction.

## 1. Introduction

Anesthetics are drugs frequently used in various medical procedures. They provide the necessary conditions for clinical interventions, such as amnesia, hypnosis, analgesia, and immobility, but can also cause cardiovascular (CV) and respiratory depression [1]. Additionally, these agents affect other organ systems, including endothelial function. Research has demonstrated that both general and local anesthetics significantly modulate the biological activity of endothelial cells under aerobic and ischemia-reperfusion conditions, making the endothelium a target for anesthetics within the CV system [2].

**Figure 1:**
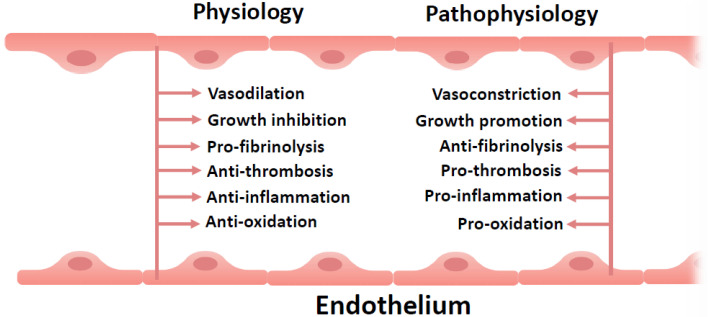
Healthy endothelium maintains a balance between opposing conditions. Endothelial cells perform paracrine-endocrine, metabolic and synthetic functions, contributing to homeostasis by maintaining balance between various processes. When damage to the endothelium occurs, the balance shifts to the predominance of the processes described on the right side of the figure.

The endothelium comprises a single layer of endothelial cells lining the vascular and lymphatic systems, covering a surface area greater than 1000 m^2^ [3]. As one of the largest paracrine and metabolic organs (e.g., the total mass of endothelial cells in an adult human exceeds that of the liver), the endothelium plays a pivotal role in vascular homeostasis. The endothelium functions as a dynamic paracrine organ that regulates vasomotor tone, coagulation, fibrinolysis, inflammation, and angiogenesis. Structurally and functionally intact endothelium maintains a balance between opposing states: vasodilation and vasoconstriction, growth inhibition and stimulation, antithrombotic and prothrombotic effects, anti-inflammatory and pro-inflammatory responses, as well as antioxidant and pro-oxidant effects (Figure 1) [4].

The physiological function of the endothelium is essential for maintaining normal blood flow and preventing the development of CV diseases. The role of endothelial dysfunction has been established in the development of chronic diseases such as atherosclerosis, arterial hypertension, chronic heart failure, diabetes mellitus, chronic obstructive pulmonary disease, chronic kidney disease, inflammatory bowel disease and others [5]. Endothelial dysfunction can be caused by various factors, including oxidative stress, chronic inflammation, and numerous other risk factors (smoking, obesity, and high blood pressure). The complex network of endothelial cells can be damaged not only following surgical procedures but also due to numerous comorbidities in patients [6,7]. Endothelial function is compromised both before and during surgery, and its trajectory in the postoperative period varies—some patients exhibit gradual recovery, while others experience persistent or even worsening dysfunction. For example, in patients undergoing minor-to-moderate surgery, endothelial function measured by reactive hyperemia index (RHI) was significantly suppressed on the day of surgery, with an average recovery by postoperative day 4; yet recovery was less pronounced in patients with comorbidities such as diabetes, obesity, and hyperuricemia, or when sevoflurane was used as the anesthetic [8]. The insufficient bioavailability of nitric oxide (NO) is widely recognized as the most significant risk factor for developing endothelial dysfunction [9]. Endothelial dysfunction is characterized by impaired endothelium-dependent vasodilation, and may also involve oxidative stress, chronic inflammation, increased leukocyte adhesion, vascular hyperpermeability, and endothelial cell aging. Additionally, increased apoptosis, the expression of intercellular adhesion molecule-1 (ICAM-1), and disrupted NO synthesis can be indicative of endothelial dysfunction [10]. Recent study has shown that altered endothelial cell metabolism and the transition of the endothelium to a mesenchymal phenotype may represent novel forms of endothelial dysfunction [11].

More recent research emphasizes the importance of investigating anesthetic effects on various organ systems, including the endothelium [2]. Understanding the mechanisms by which anesthetics influence endothelial function may be crucial for patient care and treatment. Anesthetics exert complex cardiovascular effects, including depression of cardiac contractility and arterial pressure, and may precipitate adverse events such as ischemia and arrhythmias. Certain anesthetics such as chloroform and cyclopropane can induce changes in cardiovascular function and blood pressure [12]. Basic research has demonstrated that both general and local anesthetics significantly modulate endothelial cell biological activities under both aerobic and ischemia-reperfusion conditions, making the endothelium a crucial cardiovascular target [2]. Additionally, different anesthetics may show varying effects on endothelial glycocalyx integrity, with regional anesthesia potentially offering superior endothelial protection compared to general anesthesia by more effectively inhibiting sympathetic nervous system activation and reducing systemic inflammatory responses [13]. Unfortunately, an increasing number of patients undergoing surgical procedures exhibit some degree of endothelial dysfunction, along with multiple comorbidities.

Anesthesiologists need to be familiar with the risk factors and conditions that either cause or result from endothelial dysfunction, such as hypercholesterolemia, diabetes mellitus, smoking, hypertension, atherosclerotic disease, chronic inflammatory diseases, and acute infections, as these factors may influence the effects of anesthetics or the chosen anesthetic technique [14]. Recent studies suggest that noise, air pollution, heavy metal poisoning, and other environmental factors may contribute to the development of endothelial dysfunction [15].

Given the clinical relevance of endothelial dysfunction in perioperative and cardio-vascular medicine, it is important to synthesize available evidence regarding anesthetic-related effects. The objective of this review is to provide a comprehensive overview of how various anesthetic agents influence endothelial physiology, focusing on oxidative stress, nitric oxide pathways, inflammation, vascular permeability, and glycocalyx integrity. The review further aims to discuss translational implications for anesthetic management and to outline potential therapeutic strategies that may protect endothelial function during surgery.

## 2. The Effects of Anesthetics on Endothelium-Mediated Vasodilation and Vasoconstriction

### 2.1. General Mechanisms of Endothelial Regulation

In addition to their anesthetic effects on the body, anesthetics exert a wide range of different biological effects. Particularly significant are their effects on endothelium-mediated modulation of vasoconstriction and vasodilation. As the key regulator of vascular homeostasis, the endothelium synthesizes a wide range of vasoactive substances, which are crucial for regulating vascular tone. The recognition of the importance of endothelial-derived vasoactive mediators began with the discovery of prostacyclin, a potent vasodilator. In addition to prostacyclin, the vasodilatory effects of the endothelium are mediated by NO, endothelial-derived hyperpolarizing factor (EDHF), adrenomedullin (AM), and gasotransmitters such as hydrogen sulfide (H_2_S) and carbon monoxide (CO) [16,17,18]. On the other hand, endothelium-dependent vasoconstriction is mediated by endothelins (the most potent being endothelin-1, ET-1), angiotensin II (Ang II), thromboxane (TxA2), and free radicals [16,17,18,19,20,21,22].

### 2.2. Nitrous Oxide (N_2_O)

Nitrous oxide (N_2_O), a gas used in anesthesia for decades, not only provides anesthesia and analgesia but also has the potential to induce endothelium-mediated vasodilation and vasoconstriction. Research indicates that N_2_O increases cerebral blood flow in both humans and animals, suggesting a vasodilatory effect. However, variations in administered doses, interactions with other anesthetics, and potential differences between studied species lead to inconsistent findings [23]. Hancock and colleagues showed that inhalation of 50% N_2_O results in a significant increase in cerebral blood flow and a reduction in vascular tone [24]. Conversely, an experimental study using a pig model showed an endothelial vasoconstrictor response when exposed to 70% N_2_O [25].

Research has shown that N_2_O can exert its vasodilatory effects by increasing the plasma concentration of cyclic guanosine monophosphate (cGMP) [26]. It is known that NO mediates the relaxation of vascular smooth muscle through the activation of cytosolic guanylate cyclase and the subsequent increase in cGMP levels. Therefore, N_2_O may exert its vasodilatory effects through NO. Certain studies have indicated that N_2_O combined with isoflurane results in more potent cerebral vasodilation compared to the equipotent dose of isoflurane alone [27]. Furthermore, N_2_O irreversibly inactivates the enzyme methionine synthase by oxidizing the cobalt atom in vitamin B12, leading to a reduction in methionine synthesis [28,29,30]. Decreased levels of methionine can increase plasma homocysteine concentrations [31]. Elevated serum levels of homocysteine represent a significant independent risk factor for the development of endothelial dysfunction, which may be partially explained by the inhibition of endothelial nitric oxide synthase (eNOS) [32,33]. It is known that eNOS is a crucial factor in NO synthesis, and thus N_2_O may indirectly inhibit NO-induced vasodilation.

### 2.3. Volatile Anesthetics (Halothane, Enflurane, Isoflurane, Sevoflurane, Desflurane)

Recent studies have indicated that the use of inhalational anesthetics may offer protection against the development of angiographic vasospasm in patients who have suffered from aneurysmal subarachnoid hemorrhage (SAH) [34]. In clinical practice, it is known that volatile anesthetics lead to a reduction in systemic vascular resistance and a subsequent decrease in arterial blood pressure. However, research on experimental animals has shown different results. Anesthetics such as halothane, enflurane, isoflurane, and sevoflurane have increased pulmonary artery perfusion pressure in a dose-dependent manner, leading to constriction of the pulmonary arteries. Among the tested anesthetics, halothane exhibited a significantly greater vasoconstrictor effect in all tested concentrations [35]. In a study by Krishnakumar and colleagues, increasing the minimal alveolar concentration (MAC) of desflurane from 0.8 to 1.3 over five minutes resulted in vasodilation of blood vessels [36]. These findings suggest that the effects of inhalational anesthetics on the endothelium vary depending on the concentration used. Recent experimental studies have shown that volatile anesthetics can lead to endothelial dysfunction; however, the exact mechanism of this effect is not yet fully understood. In an experimental study on Wistar rats, it was shown that sevoflurane impairs vasoconstriction of aortic rings, while isoflurane did not exhibit such an effect. The results of this study suggest that reduced production of NO and cytokines, as well as increased activity of matrix metalloproteinase 2 (MMP2), may be involved in vascular dysfunction following sevoflurane anesthesia [37]. Previous studies have failed to confirm that the vasodilatory effect of inhalational anesthetics is due to increased NO release from endothelial cells [38,39]. Experimental studies have also shown that inhalational anesthetics may reduce the formation of reactive oxygen species (ROS) [40]. It is possible that inhalational anesthetics indirectly prolong the vasodilatory effects of NO. However, the exact mechanism of the vasodilatory effects of inhalational anesthetics remains under investigation. Older studies suggest that volatile anesthetics promote endothelium-independent relaxation of smooth muscle by reducing intracellular Ca^2+^ availability, or by decreasing the sensitivity of contractile proteins to Ca^2+^ [41,42]. Experimental study on rabbits suggests that desflurane reduces coronary vascular resistance through the simultaneous release of prostaglandins and NO [43]. Other experimental studies have reported that inhalational anesthetics may impair vasorelaxation by interfering with EDHF production, linked to cytochrome P450 activity [44]. Another possible mechanism of vasodilation mediated by inhalational anesthetics is the reduction in ET-1 levels, a potent vasoconstrictor. An experimental study conducted on rats showed that isoflurane and enflurane, depending on the concentration used, significantly reduce ET-1-induced contraction of isolated rat aorta [45].

As previously demonstrated, the effects of volatile anesthetics on endothelium-dependent vasodilation and vasoconstriction are multifaceted and complex, involving modulation of vasoactive substances and intracellular signaling pathways. Understanding the effects of volatile anesthetics on the endothelial function of experimental animals may have significant translational implications.

### 2.4. Intravenous Anesthetics

#### 2.4.1. Propofol

On the other hand, intravenous anesthetics, predominantly exhibit vasodilatory effects, leading to a decrease in arterial blood pressure upon the initiation of anesthesia. It is well known that propofol, the most frequently used anesthetic, is a potent venous vasodilator. In this manner, propofol reduces blood flow to the right side of the heart, consequently leading to a decrease in cardiac output. Propofol exerts some of its effects by increasing levels of gaseous transmitters such as NO and CO [46,47,48]. Additionally, the possibility that propofol exerts its vasoactive effects through H_2_S cannot be excluded [22,49]. Some researchers believe that propofol achieves its cardioprotective effects by increasing levels of eNOS and/or NO. The mechanisms through which propofol exerts its potent vasodilatory effects include reduction in sympathetic tone, endothelium-independent vasodilation mediated by Ca^2+^ ions, action on Ca^2+^-activated K^+^ channels (BKCa channels), and transient receptor potential ankyrin 1 channel (TRPA1) [50,51,52,53,54]. Propofol also affects K-ATP channels in the sarcolemma of cardiomyocytes, which are normally closed under physiological conditions but provide protection against cardiac ischemia when activated [55,56]. Some studies suggest that propofol may bind to specific sites on actin and myosin, reducing the response of myofilaments to Ca^2+^ [57]. Based on available research, it can be concluded that the cardioprotective effects of propofol are likely due to its non-anesthetic cardiac and vasodilatory effects, which are probably mediated through NO, H_2_S, CO, as well as through the regulation of Ca^2+^ influx [49,58].

#### 2.4.2. Ketamine

Although ketamine belongs to the group of intravenous anesthetics, it exhibits a unique mechanism of action that distinguishes it from other anesthetics such as propofol. Its effects on the heart and blood vessels are characterized by sympathetic stimulation and parasympathetic inhibition, resulting in increased arterial blood pressure and heart rate [59]. The mechanisms of ketamine’s cardiovascular effects include blockade of cardiac vagal nerves, increased release of catecholamines, and stimulation of vasoconstriction through activation of alpha-adrenergic receptors. This combined action of ketamine leads to increased venous return to the heart, explaining the characteristic hemodynamic changes associated with its use [60,61].

#### 2.4.3. Etomidate

The third frequently used intravenous anesthetic in cardiac anesthesia is etomidate. This drug provides optimal cardiovascular stability, which is explained by the stimulation of central adrenergic receptors, with the absence of myocardial depression and minimal changes in heart rate or arterial blood pressure [61,62]. However, the impact of etomidate on the vascular system is complex and dose-dependent. Etomidate demonstrates the ability to inhibit endothelial responses to factors such as NO and EDHF, indicating an increase in vascular tone through the inhibition of vasodilatory mechanisms. On the other hand, under experimental conditions, etomidate has inhibited norepinephrine-induced vasoconstriction, suggesting a direct vasodilatory effect of this anesthetic [63,64]. Furthermore, earlier studies indicate that etomidate and thiopental selectively reduce EDHF-mediated vasodilation without affecting vasoconstriction, thereby modifying the response of coronary microcirculation to bradykinin [65].

#### 2.4.4. Benzodiazepines (Midazolam)

Benzodiazepines are widely used drugs for inducing sedation in intensive care units and emergency medicine, with short-acting midazolam being the drug of choice due to the rapid recovery of patients following its administration [66]. Experimental data indicate that midazolam exerts a dual effect on blood vessels depending on the concentration. At low concentrations, midazolam predominantly induces endothelium-mediated vasodilation, while at high concentrations, it results in endothelium-independent vasodilation, characterized by reduced sensitivity of aortic rings to Ca^2+^ [67].

### 2.5. Opioids

Opioids play a significant role in various areas of medicine, particularly in pain management. Their use ranges from adjuvant agents in anesthesia to primary agents during surgical procedures and postoperative care [68]. The effect of opioid analgesics, such as morphine and fentanyl, on endothelial function is complex. Research suggests that opioid analgesics may induce vasodilation through modulation of NO synthesis, with this effect primarily achieved through the central nervous system [69]. Opiates, such as morphine, are commonly used as the analgesics of choice for managing severe pain episodes; however, many opioids possess strong histaminergic properties. Following administration, patients may experience pruritus, vasodilation, bronchoconstriction, and urticaria due to histamine release and elevated histamine levels in the blood after oral morphine administration may contribute to an increased risk of acute chest syndrome [70].

### 2.6. Local Anesthetics

It is not excluded that some local anesthetics also exert endothelium-mediated vasoactive effects. One study confirmed that ropivacaine, a local anesthetic, induces vasoconstriction in the mesenteric arteries of mice. This vasoconstrictive effect of ropivacaine is partially mediated through the endothelium and cyclooxygenase pathways [71]. The standard clinical dose of bupivacaine, induces vasodilation, while a lower dose shows a vasoconstrictive effect [72]. In clinical practice, local anesthetics are often administered in combination with vasoconstrictors, achieving inhibition of endothelium-mediated vasodilation and, indirectly, narrowing of blood vessels [73]. The aforementioned vasoconstrictive effect of local anesthetics could potentially lead to localized endothelial dysfunction. However, further research is necessary regarding the endothelium-mediated vasoactive effects of local anesthetics.

Additionally, different anesthesia techniques may exhibit varying effects on endothelial function, primarily through modulation of the sympathetic nervous system. Spinal and epidural anesthesia can indirectly affect endothelial function through the regulation of sympathetic activity. These anesthesia techniques have the potential to induce changes in vascular tone and endothelial function, which can be attributed to their impact on the autonomic nervous system [12].

In summary, anesthetic agents exhibit distinct endothelial and vascular effects, de-pending on drug class, dose, and clinical context. While propofol and volatile anesthetics generally promote vasodilation, ketamine and some local anesthetics can induce vasoconstriction. These distinct pharmacological profiles underscore the critical importance of individualized anesthetic selection to optimize endothelial protection in perioperative care.

## 3. Effect of Anesthetics on Endothelium-Mediated Inhibition and Stimulation of Growth Factors

### 3.1. General Mechanisms of Growth Factor Regulation

Although the primary effect of anesthetics is to achieve anesthesia in patients during surgical procedures, recent research increasingly emphasizes their impact on various cellular signaling pathways and non-anesthetic effects. Under physiological conditions, functionally intact endothelium allows for a balance between inhibition and activation factors of growth [4,21]. Adequate regulation of endothelial function and growth factors plays a crucial role in regulating the structure of blood vessels, maintaining tissue homeostasis, and wound healing [4]. Therefore, a detailed investigation of the complex relationship between anesthetics and endothelial cells is necessary, with a specific focus on their effects on the modulation of growth factors.

### 3.2. Propofol and Growth Factors

It is believed that propofol can affect endothelium-mediated growth factors. Research has shown that propofol can reduce levels of interleukin-6 (IL-6), interleukin-8 (IL-8), vascular endothelial growth factor (VEGF), and prostaglandin E2 (PGE2). In this way, propofol potentially inhibits tumor growth and metastasis by decreasing the production of growth factors [74]. Some studies have indicated that certain anesthesia techniques can influence the levels of substances produced by the endothelium. Specifically, anesthesia techniques may affect serum concentrations of vascular endothelial growth factor C (VEGF-C) and transforming growth factor β (TGF-β), thereby impacting angiogenesis. In a study by Looney and colleagues, after surgery in patients who underwent regional paravertebral block with propofol sedation, significantly lower levels of VEGF-C were observed compared to patients who underwent general anesthesia [75]. VEGF-C is considered an important factor in tumor-induced angiogenesis and the development of distant metastases. Therefore, this research demonstrates that certain anesthesia techniques play a significant role in reducing the risk of tumor progression or recurrence after surgery.

### 3.3. Volatile Anesthetics and Growth Factors

Certain volatile anesthetics, such as isoflurane, sevoflurane, and desflurane, may be involved in the modulation of growth factors crucial for endothelial function. Isoflurane is thought to exert cardiovascular protective effects through VEGF factors [76]. VEGF is known to contribute to the synthesis of NO via eNOS. A study exploring the complex relationship between isoflurane and the endothelium found that isoflurane increases eNOS activity, which may be a significant mechanism behind this anesthetic’s cardioprotective effect [77]. Previous research has documented that isoflurane can increase VEGF factor levels, potentially promoting the growth and spread of tumor cells. Elevated VEGF levels have been observed in kidney cancer cells exposed to isoflurane, suggesting a potentially harmful effect of this anesthetic. It is also suggested that the effects of isoflurane on VEGF are mediated through hypoxia-inducible factors (HIF), which play a crucial role in the response to low oxygen conditions and the progression of malignant diseases [78]. Furthermore, sevoflurane, a volatile anesthetic more commonly used in clinical practice, can enhance the growth and proliferation of human endothelial progenitor cells similar to stem cells [79]. These cells, involved in tissue repair and regeneration, play a crucial role in modulating VEGF levels. The impact of sevoflurane on these processes is noteworthy, as it may enhance perioperative vascular healing through its effects on VEGF. On the other hand, the inhalational anesthetic N_2_O can reduce VEGF levels. Research indicates that VEGF levels are significantly elevated at the onset of surgery compared to subsequent time points, suggesting that the timing of anesthetic administration plays an important role in affecting endothelial growth factors [80].

### 3.4. Intravenous Anesthetics and Growth Factors

Intravenous anesthetics have been demonstrated to exert significant effects on endothelial growth factors. Total intravenous anesthesia (TIVA), typically comprising a combination of propofol and remifentanil, has been shown to effectively suppress the release of VEGF-C. In contrast, the inhalational anesthetic sevoflurane does not exhibit this inhibitory effect. Research suggests that TIVA may attenuate surgery-induced VEGF-C release, while its impact on TGF-β levels appears to be minimal [81]. Furthermore, Galos et al. reported that intravenous administration of lidocaine during breast cancer surgery resulted in reduced postoperative expression of extracellular neutrophil traps (NETosis) and matrix metalloproteinase 3 (MMP3), potentially contributing to a decrease in disease recurrence [82].

These findings underscore the complex interplay between various anesthetic agents and endothelial-mediated growth factors. The effects of anesthetics on these factors involve a nuanced balance of inhibition and stimulation, with some anesthetics suppressing angiogenesis while others demonstrate stimulatory effects. This complexity highlights the need for further research to elucidate the mechanisms of anesthetic action and their potential impacts on growth factors. Such investigations are crucial for minimizing adverse effects on vascular homeostasis and tissue repair processes.

## 4. Effects of Anesthetics on Endothelial-Mediated Antithrombotic and Prothrombotic Functions

### 4.1. General Mechanisms

The structural and functional integrity of the endothelium is fundamental to the maintenance of vascular homeostasis. Endothelial dysfunction can disrupt the delicate equilibrium between procoagulant and anticoagulant mediators, potentially leading to thrombosis [4].

Anesthetic agents commonly employed in clinical practice have been shown to exert diverse effects on endothelial function, potentially acting as either prothrombotic or antithrombotic factors. This complex interaction requires a thorough investigation of the relationship between anesthetics and endothelial function, with particular emphasis on their impact on thrombosis regulation. Anesthesia, while indispensable for the safe execution of surgical procedures, has the potential to modulate endothelial function and, consequently, influence thrombotic processes. Therefore, elucidating the mechanisms by which various anesthetic agents affect endothelial-mediated antithrombotic and prothrombotic functions is of significant clinical and research interest.

### 4.2. Propofol

The impact of propofol on platelet aggregation remains a topic of debate, with various studies providing inconsistent results. One study suggests that propofol exhibits inhibitory effects on platelet aggregation by modulating pro-inflammatory lipid mediators without altering intracellular Ca^2+^ levels, implying a potential influence on the endothelial inflammatory response through Ca^2+-^independent mechanisms [83]. These findings indicate that propofol’s effects are likely mediated through signaling pathways that are not yet fully elucidated. Additionally, the same study demonstrated a dose-dependent effect of propofol on endothelial function, suggesting that varying concentrations of this anesthetic may differentially impact inflammation and platelet activation. However, more recent studies have yielded contrasting results. An in vitro study conducted by Chung and colleagues reported that propofol, when administered in concentrations required for sedation and general anesthesia, does not significantly affect platelet aggregation [84]. These findings support the safety profile of propofol for use in surgical interventions.

### 4.3. Volatile Anesthetics

Son and colleagues examined the effects of different anesthetics (propofol-remifentanil TIVA and inhalational anesthesia with sevoflurane) on platelet function. The study demonstrated that the use of TIVA during surgery significantly reduced platelet aggregation induced by collagen and ADP, while the administration of sevoflurane did not show such an effect [85]. Additionally, inhalation of low doses of sevoflurane (<1 vol % end-tidal) inhibits granulocyte-platelet interactions induced by various agonists (including ADP, arachidonic acid, and TRAP-6) for up to 24 h post-administration, thereby counteracting thromboinflammatory processes [86].

### 4.4. Local Anesthetics

The antithrombotic properties of local anesthetics are also well-documented in clinical practice. In addition to the reversible blockade of Na^+^ channels in the axons of nerve cells, local anesthetics stabilize platelet membranes and inhibit the release of α-granules, platelet aggregation, and the TxA2 signaling pathway [87]. Dregalla and colleagues showed that the application of bupivacaine leads to a reduction in platelet surface area, dysregulation of intracellular Ca^2+^, increased ROS production in mitochondria, and apoptosis of platelets. In contrast, lidocaine and ropivacaine had minimal effects on platelet metabolism and viability [88].

Furthermore, clinical evidence suggests a beneficial effect of neuraxial anesthesia (which involves the use of local anesthetics) in the prevention of deep vein thrombosis (DVT) in patients undergoing high-risk surgical interventions [89]. Although the mechanism behind this preventive effect of local anesthetics is not fully understood, it is hypothesized that there is an interaction between anesthetics and immune cells (such as monocytes). Additionally, the authors of the previous study propose that adhesion molecules associated with monocyte-platelet aggregate formation, as well as the substance P-neurokinin 1 receptor (SP/NK1R) signaling pathway, are key factors. Local anesthetics and NK1R antagonists may potentially prevent venous thrombotic disorders in perioperative settings.

It is crucial to recognize that surgical interventions, depending on their nature and anatomical location, can exert prothrombotic effects on the body. Consequently, the selection of an appropriate anesthetic agent and anesthesia technique with antithrombotic properties is of paramount importance, particularly for high-risk surgical patients.

### 4.5. Ketamine

While the majority of anesthetic agents demonstrate antithrombotic effects, some, such as ketamine, may exhibit prothrombotic properties. Ketamine’s mechanism of action involves stimulation of the sympathetic nervous system, leading to systemic catecholamine release [61]. This sympathetic activation could potentially result in endothelial activation and subsequent thrombus formation. However, it is important to note that the current body of evidence is insufficient to conclusively establish the prothrombotic effects of ketamine. Further research is warranted to elucidate its precise impact on coagulation cascades and vascular function.

## 5. The Effect of Anesthetics on the Anti-Inflammatory and Pro-Inflammatory Roles Mediated by the Endothelium

### 5.1. General Mechanisms of Endothelial Inflammation

Endothelial cells play a crucial role in the onset and progression of vascular inflammation, which is associated with the etiology of numerous diseases, including cardiovascular diseases, respiratory conditions, atherosclerosis, and diabetes mellitus. Activated endothelial cells release potent inflammatory mediators that stimulate the immune system and contribute to the development of inflammatory states. Endothelial dysfunction represents an initial step in the pathogenesis of vascular inflammatory diseases [90].

In the context of regulating inflammatory processes, the role of NO stands out. Under physiological conditions, NO predominantly exhibits anti-inflammatory properties by inhibiting leukocyte adhesion processes and by suppressing the expression of adhesion molecules such as ICAM-1 and vascular cell adhesion molecule 1 (VCAM-1) [91]. However, in pathological conditions, significant alterations in NO metabolism and function occur, where excessive NO synthesis can lead to tissue damage, which is especially pronounced in inflammatory autoimmune diseases. These findings highlight the dual role of NO in the regulation of inflammation [92].

The anti-inflammatory properties of the endothelium can be partly attributed to the production of prostacyclin. Prostacyclin plays a key regulatory role in the cardiovascular system, contributing to the relaxation of vascular smooth muscle cells and the inhibition of platelet aggregation. Furthermore, prostacyclin plays a crucial role in modulating the inflammatory response through its capacity to inhibit leukocyte adhesion and suppress vascular smooth muscle cell proliferation [93,94].

In contrast to the endothelium’s physiological anti-inflammatory role, pathological conditions induce significant modifications in the endothelial phenotype, creating an environment conducive to the development of inflammation, vasoconstriction, and thrombosis. During inflammatory processes of diverse etiologies, there is a marked increase in the synthesis of pro-inflammatory mediators, including a range of cytokines such as interleukin-1 (IL-1), IL-6, interleukin-18 (IL-18), tumor necrosis factor-alpha (TNF-α), and C-reactive protein (CRP). These mediators induce the formation of a pro-inflammatory endothelial phenotype, characterized by increased expression of E-selectin, VCAM-1, ICAM-1, and monocyte chemoattractant protein-1 (MCP-1) on the endothelial surface [95,96,97].

### 5.2. Volatile Anethetics

Given the significance of endothelial cells in regulating inflammation and maintaining vascular homeostasis, it is essential to investigate the impact of anesthetics on endothelial function and the modulation of inflammatory responses. Studies suggest that anesthetics possess a dual role, exhibiting both pro-inflammatory and anti-inflammatory effects [98,99]. Inhalation anesthetics, such as sevoflurane, desflurane, and isoflurane, demonstrate significant anti-inflammatory properties by inhibiting the activation of nuclear factor kappa B (NF-kB), a key transcription factor in regulating the inflammatory response, thus reducing cytokine production [100]. On the other hand, halogenated inhalation anesthetics inactivate glycogen synthase kinase-3β, a critical enzyme involved in cellular injury and systemic inflammatory response syndrome (SIRS). Concurrently, halogenated inhalation anesthetics disrupt NF-kB activation, further contributing to their anti-inflammatory properties [101].

### 5.3. Local Anesthetics

In addition to inhalation anesthetics, local anesthetics also exhibit significant anti-inflammatory properties, which can be crucial in clinical conditions associated with chronic inflammation. A study by Weinschenk and colleagues demonstrated that local anesthetics can alleviate the inflammatory response induced by lipopolysaccharides by reducing the synthesis of the cytokine TNF-α. These findings highlight the potential role of local anesthetics in the treatment of chronic inflammatory conditions [102].

### 5.4. Propofol

**Figure 2:**
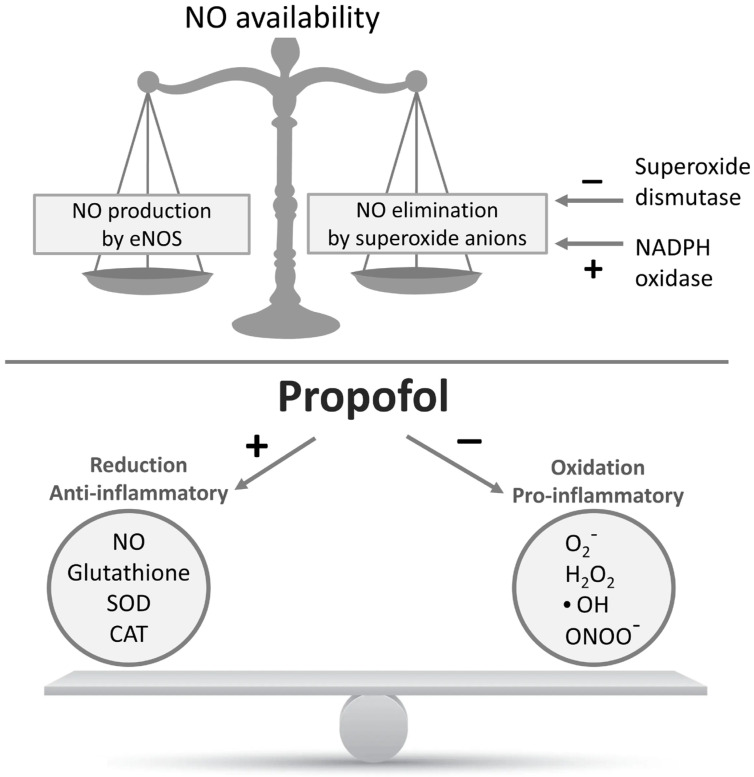
Homeostatic balance between reactive oxygen species and reactive nitrogen species production in endothelium determine NO availability (above). The proposed cardioprotective effects of anesthetic propofol on decreased production of reactive oxygen species and pro-inflammatory factors, and increased production of NO and anti-inflammatory factors (below). Symbols ‘+’ and ‘-‘ indicate stimulation/increase and inhibition/decrease of the depicted effects, respectively. Abbreviations: NO—nitric oxide, O_2_^−^—superoxide radical, •OH—hydroxyl radical, H_2_O_2_—hydrogen peroxide, ONOO^−^—peroxynitrite, eNOS—endothelial nitric oxide synthase, NADPH oxidase—nicotinamide adenine dinucleotide phosphate oxidase, SOD—superoxide dismutase, CAT—catalase.

Furthermore, propofol exerts anti-inflammatory effects by inhibiting NF-kB activation, partly through reduction in ROS-mediated Akt signaling (Figure 2) [103].

### 5.5. Intravenous Anesthetics

Intravenous anesthetics, including ketamine and thiopental, have been observed to modulate the release of anti-inflammatory cytokines. This anti-inflammatory effect is particularly noteworthy in severe clinical conditions such as endotoxemia, a frequent complication encountered in intensive care settings [104]. However, the impact of ketamine on the inflammatory response is multifaceted and not fully elucidated, with current literature presenting conflicting findings. While some investigations have emphasized ketamine’s anti-inflammatory properties, others have suggested its potential to elicit an inflammatory response. The effect of ketamine on the expression of proinflammatory cytokines, such as TNF-α, appears to be dose-dependent and influenced by the duration of administration [105,106].

### 5.6. Clinical Considerations and Anesthesia Techniques

Recent studies suggest that under certain conditions, anesthetics may exhibit both pro-inflammatory and immunomodulatory properties. In cases of prolonged use, high doses, or the presence of immunodeficiency, these properties of anesthetics may increase the risk of opportunistic infections and lead to immunosuppression [99]. Additionally, some anesthetics are believed to impair various immune system functions, either directly by disrupting immune cell function or indirectly by modulating the body’s stress response [107,108].

However, in addition to the type of anesthetic used, it is well-known that various factors contribute to immunomodulation and the regulation of the inflammatory response. Surgical trauma and mechanical ventilation can trigger inflammation, making it difficult to distinguish their effects from those of the anesthetics [109]. Different anesthesia techniques can significantly influence the systemic inflammatory response. TIVA in combination with propofol, reduces CRP levels, which can have a beneficial effect on postoperative outcomes [110].

In addition to the type of anesthetic and surgical intervention, the individual characteristics of patients also play a significant role in modulating the immune response. The presence of chronic diseases or obesity is often accompanied by chronic inflammation, further complicating the patient’s immune response and potentially negatively affecting the outcome of anesthesia and surgical treatment. Both inhalational and intravenous anesthesia can further disrupt immune system homeostasis, leading to the activation of immune cells and the release of pro-inflammatory and anti-inflammatory cytokines, resulting in increased cellular adhesion and excessive ROS production [111].

Understanding the interaction between anesthetics and endothelial-mediated inflammatory responses has significant clinical implications. Appropriate selection of anesthetics based on their effects on the endothelium can optimize perioperative management, reduce inflammatory responses, and improve treatment outcomes. Future research should focus on investigating the impact of various doses and timing of anesthetic administration on endothelial-mediated inflammatory processes to enhance perioperative care and clinical outcomes.

## 6. Effects of Anesthetics on Endothelial-Mediated Antioxidant and Prooxidant Roles

### 6.1. General Mechanisms of Oxidative Stress and Endothelial Function

The endothelium plays a crucial role in maintaining vascular homeostasis by balancing prooxidative and antioxidative processes. Oxidative stress is defined as an imbalance between these processes, resulting from weakened antioxidative defense or excessive production and accumulation of ROS [112]. Decreased synthesis of NO or increased ROS production contributes to endothelial dysfunction and subsequent vascular remodeling, platelet aggregation, impaired vasodilation, and induction of inflammation [113].

### 6.2. Propofol

Anesthetics can significantly influence endothelial function through the modulation of oxidative stress. Intravenous anesthetic propofol is well known for its pronounced antioxidant properties, attributed to the presence of a phenolic hydroxyl group in its chemical structure, similar to that of vitamin E, a well-known antioxidant. This structure enables propofol to effectively neutralize ROS, thereby contributing to the reduction in oxidative stress and endothelial protection. Due to the antioxidant effects of propofol, the potential for oral formulation of this drug is being actively explored. However, the poor bioavailability of propofol when administered orally remains a significant challenge [114,115].

The antioxidant effect of propofol is achieved through the enhancement of NO synthesis and other antioxidant enzymes, such as glutathione, superoxide dismutase (SOD), and catalase (CAT). These properties of propofol contribute to its cardioprotective effect by reducing oxidative stress and protecting endothelial-dependent vasodilation [116,117,118].

**Figure 3:**
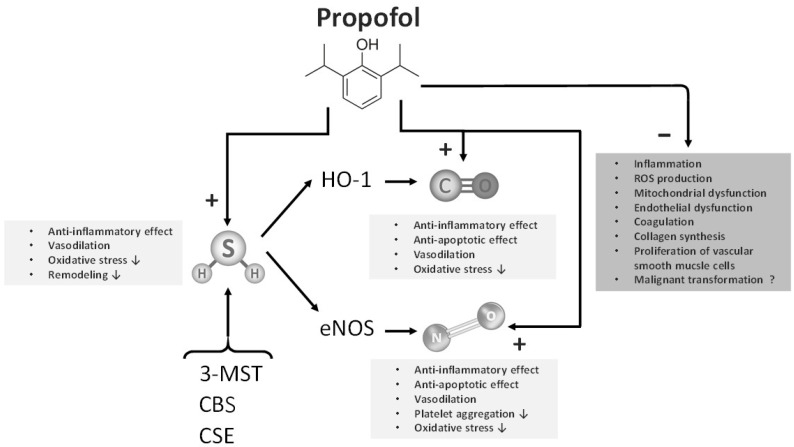
The presumed effects of propofol on the synthesis of gaseous transmitters (NO, H_2_S, NO), along with the depiction of key enzymes involved in the synthesis of NO (eNOS), H_2_S (CSE, CBS, 3-MST) and CO (HO-1). For each listed gaseous transmitter, potential effects on the cardiovascular system are described, while on the right side of the diagram, possible pleiotropic and adverse effects of propofol are illustrated. Arrows pointing downward (↓) indicates inhibition/decrease, in the described biological processes or effects; ‘+’ indicates enhancement or promotion of the effect; ‘-’ indicates inhibition or reduction of the effect; ‘?’ indicates uncertain or unclear relationship. Abbreviations: NO-nitric oxide, CO—carbon monoxyde, H_2_S—hydrogen sulfide, eNOS—endothelial nitric oxide synthase, CSE—cystathionine γ-lyase, CBS—cystathionine β-synthase, 3-MST—3-mercaptopyruvate sulfurtransferase, HO-1—heme-oxygenase 1; ROS—reactive oxygen species.

Furthermore, propofol protects against oxidative damage caused by peroxides, including exogenously applied H_2_O_2_, by increasing the antioxidant capacity of human plasma [119,120,121,122,123]. Additionally, propofol affects the reduction in metabolic O_2_ and glucose consumption in the brain, inhibits respiratory activity in neutrophils, influences the efflux and re-uptake of glutamate, and leads to the inhibition of NMDA (N-methyl-D-aspartate) receptors [124]. In vivo, propofol reduces the production of superoxide radicals (O_2_^−^) in vascular endothelial cells, protecting them from damage [125,126]. The antioxidant properties of propofol, as previously discussed, appear to be mediated, at least in part, by gasotransmitters (Figure 3).

Gonzalez et al. demonstrated that propofol modulates NO synthesis by stimulating the constitutive isoform of NOS while inhibiting the inducible isoform of the enzyme [127]. In astroglial cells, propofol reduces peroxynitrite-induced cytotoxicity and apoptosis, partly through the activation of heme oxygenase which increases CO levels [128]. Beyond antioxidant effects, propofol shows immunomodulatory properties: In vitro study by Salo and colleagues demonstrated that propofol alters the balance between T helper lymphocyte subpopulations, promoting a Th1-dominant immune response without causing generalized T helper cell suppression [129].

### 6.3. Intravenous Anesthetics

Although intravenous anesthetics are known for their predominantly antioxidant properties, study results are often inconsistent. According to certain experimental models, ketamine is superior to etomidate regarding antioxidant potential [130]. On the other hand, in an experimental study by De Oliveira and colleagues, sub-anesthetic doses of ketamine caused a significant increase in thiobarbituric acid reactive substances (TBARS), which are markers of lipid peroxidation. Additionally, these doses of ketamine led to a significant reduction in the activity of key antioxidant defense enzymes, such as superoxide dismutase (SOD) and catalase (CAT) [131]. Furthermore, thiopental, an intravenous barbiturate anesthetic, has demonstrated the ability to directly neutralize ROS, including superoxide (O_2_^−^), hydroxyl radical (•OH^−^), and NO [132]. However, when administered in sub-anesthetic and anesthetic doses, thiopental can lead to increased oxidative stress in the brain’s striatum, likely through reduced adrenaline production, resulting in systemic hypotension and an increased risk of severe complications [133]. These findings suggest opposing antioxidant and prooxidant properties of thiopental, emphasizing the importance of careful monitoring and dosing of this anesthetic in clinical practice.

### 6.4. Opioids

Despite its significance, the current research on the potential link between opioid analgesics and oxidative stress is relatively limited. Morphine, one of the most potent representatives of the opioid analgesic class, is widely used in the treatment of severe acute and chronic pain conditions. However, long-term use of morphine is associated with the development of addiction, tolerance, and multiple adverse effects. Certain studies suggest the role of oxidative stress in the induction and progression of these adverse effects of morphine. Some studies indicate that morphine induces the generation of ROS and reactive nitrogen species (RNS), while simultaneously inhibiting the antioxidant defense system [134]. On the other hand, Almeida and colleagues reported that morphine, at regular analgesic doses, contributes to reducing oxidative stress in brain glial cells [135]. Due to the different experimental study protocols, it is challenging to assess the relationship between morphine and oxidative stress, which may depend on dose, duration of exposure, and cell type [136]. Furthermore, it has been shown that tramadol, another representative of the opioid analgesic class, exerts direct toxic effects on the cardiovascular system. In an experimental study, tramadol induced an increase in myocardial injury biomarkers, creatine kinase, and myoglobin, along with cardiomyocyte degeneration, sarcomere disorganization, and interstitial collagen accumulation. Additionally, tramadol therapy increased ROS, inflammation, and endothelial dysfunction [137].

### 6.5. Inhalation Anesthetics and Local Anesthetics

Regarding inhalational anesthetics, one study has shown that sevoflurane reduces markers of lipid peroxidation and significantly increases levels of the antioxidant ceruloplasmin. It is believed that sevoflurane has a more pronounced antioxidant potential compared to propofol [138].

Local anesthetics also exhibit an important antioxidant role. Experimental studies have demonstrated that lidocaine and procaine protect the endothelium from ROS-mediated damage, preserving vascular dilation [139].

## 7. Effects of Anesthetics on Endothelial Glycoalyx

The luminal surface of all vascular endothelial cells is covered by the endothelial glycocalyx (eGCX), which comprises membrane-bound negatively charged proteoglycans, glycoproteins, glycolipids and glycosaminoglycans, which has a significant role in maintaining vascular permeability, microcirculation and homeostasis of blood vessels. It is known that in inflammatory conditions the endothelial glycocalyx can be damaged, which consequently leads to increased vascular permeability, tissue edema, increased leukocyte adhesion, increased platelet aggregation and dysregulation of vasodilation [140]. In the perioperative period, ischemia-reperfusion injury, oxidative stress, hypervolemia, and systemic inflammatory response may be responsible for glycocalyx degradation [141]. According to the classic Starling’s principle, fluid flux across the endothelium was thought to be governed by the balance between hydrostatic and oncotic pressure gradients, with reabsorption into the circulation. However, later work demonstrated that fluid return occurs predominantly through lymphatic drainage, with the eGCX playing a critical role as a molecular sieve that maintains low protein concentrations beneath it. Evidence from enzymatic removal of eGCX components shows increased permeability, albumin loss, and edema, indicating that the eGCX is a major regulator of vascular permeability [141]. More recent studies have mainly compared the effects of intravenous anesthetics (most commonly propofol) and inhalational anesthetics during operations involving ischemia-reperfusion injury. A comparison of two anesthesia techniques (propofol-remifentanil vs. sevoflurane-remifentanil) showed that the increase in serum syndecan-1 (one of the glycocalyx damage biomarkers which regulates the expression of enzymes in heparan sulfate biosynthesis and proteoglycan profile) one hour after surgery was significantly lower in the propofol group compared to the sevoflurane group [142]. Such research may indicate that propofol protects the glycocalyx significantly better than sevoflurane. In a pilot study that also compared the effects of propofol vs. sevoflurane, followed by syndecan-1 and thrombomodulin. The results show that the release of thrombomodulin (a marker of endothelial damage) was lower in the propofol group [143]. These findings may suggest that propofol anesthesia techniques may have an advantage in endothelium preservation over other techniques. Contrary to the above-mentioned study results, some studies also indicate the advantages of sevoflurane compared to propofol in certain circumstances. In patients who underwent cardiopulmonary bypass, there was significant damage to the glycocalyx as a consequence of the bypass, but the degradation of the glycocalyx was less pronounced in the sevoflurane group [144]. These findings suggest that sevoflurane may reduce cardiopulmonary bypass-induced glycocalyx damage compared to propofol. Comparisons of propofol with other inhaled anesthetics, such as desflurane, were also made. No differences were found in changes in syndecan-1 levels [145]. In addition to studies on surgical patients, the results on experimental models of sepsis comparing intravenous and inhalation sedation with propofol and isoflurane are certainly interesting. In this study, microvascular flow, glycocalyx thickness, and syndecan-1 were measured in septic and healthy mice. In healthy animals, propofol maintained microvascular flow better than isoflurane. Also, measuring the thickness of the glycocalyx showed that the glycocalyx was significantly thinner (more damaged) in the sepsis-isoflurane group compared to the sepsis-propofol group [146]. These animal findings may also complement clinical knowledge and suggest potential protective properties of propofol on the endothelium. It can be concluded that the intravenous anesthetic propofol in certain situations protects the endothelial glycocalyx better than volatile anesthetics (sevoflurane, isoflurane, desflurane), especially in conditions of ischemia-reperfusion injury, minor surgical stress or sepsis. On the other hand, with a more severe systemic burden such as cardiac surgery, some inhaled anesthetics such as sevoflurane show an advantage in protecting the glycocalyx. Unfortunately, many studies have not found a significant difference in the effects of anesthetics on the glycocalyx, suggesting that the effects may depend on the dosage, duration and type of surgery and the inflammatory response present. When it comes to local anesthetics and regional anesthesia techniques, effects on endothelial function and the glycocalyx may also be important. Lidocaine is known as a drug with anti-inflammatory properties that can potentially modulate the inflammatory response. Some animal studies indicate that lidocaine may protect the glycocalyx possibly by reducing inflammation and inhibiting the release of cytokines, histamines, and proteases that damage the glycocalyx. In an experimental pig lung reperfusion model, lidocaine premedication reduced the increase in plasma syndecan-1 and heparan sulfate and attenuated the increase in matrix metalloproteinase 9 and heparinase, indicating less damage to the glycocalyx [147]. Unfortunately, these results applied to the human population are modest. In a pilot study conducted on patients undergoing major abdominal surgery, continuous intravenous lidocaine administration during surgery vs. placebo (saline solution), there was no significant difference between the lidocaine and placebo groups regarding the degree of increase in syndecan-1 [148]. The application of neuraxial anesthesia (spinal, epidural) potentially avoids some harmful effects of general anesthesia. Research findings comparing neuraxial versus general anesthesia indicate that regional anesthesia damages the glycocalyx less than general anesthesia [13]. This finding may be explained by the fact that regional anesthesia reduces systemic stress and increases the need for higher volume infusions, which may preserve the endothelial barrier. Finally, experimental evidence suggests that avoiding fluid overload or accumulation, use of volatile anesthesia, maintaining normoglycemia, and maintaining normal plasma albumin levels, and several agents like hydrocortisone, hyaluronan, albumin, and sulodexide can protect against endothelial glycocalyx damage [149]. These results suggest that certain drugs and anesthesia techniques can contribute to the preservation of endothelial functions; however, further research is needed to confirm the advantage of certain anesthetics and anesthesia techniques compared to other drugs and methods.

## 8. Ferroptosis and Anesthetic Agents in Endothelial Function

Ferroptosis, an iron-dependent form of regulated cell death, is closely related to iron and lipid metabolism and can be triggered by various physiological conditions and pathological stresses. This distinct cell death pathway connects oxidative stress and inflammatory responses through iron and lipid metabolism, thereby playing a pathological role in the development of cardiovascular diseases and other diseases [150]. Evidence indicates that ferroptosis in endothelial cells contributes to various pathological processes, including stroke, atherosclerosis, and cancer. Understanding the molecular mechanisms of ferroptosis and its involvement in endothelial cell dysfunction across different disease states has important implications for anesthetic management and perioperative endothelial protection [151,152].

Recent evidence suggests that anesthetic agents may modulate ferroptosis pathways in endothelial cells. Experimental data suggests that propofol can mitigate iron overload-induced oxidative stress and apoptosis by modulating iron metabolism proteins (ferritin, iron import transferrin receptor 1, ferroportin 1) and suppressing inflammatory signaling, while also activating protective pathways such as nuclear factor erythroid 2-related factor/glutathione peroxidase 4 (Nrf2/GPX4). However, at higher concentrations propofol may exert neurotoxic effects, promoting ROS accumulation, and cognitive dysfunction. These conflicting results indicate that overall impact of propofol on ferroptosis mediated injury remains to be fully clarified [153].

On the other hand, ketamine appears to exert rapid antidepressant effects in chronic restrained stress treated (CRS) rats partly by inhibiting ferroptosis, as evidenced by increased FTH1 and GPX4 expression and reduced Tfr1 levels. These findings suggest that modulation of ferroptosis in the habenular nucleus may represent a key mechanism underlying ketamine’s antidepressant action [154].

Additionally, volatile anesthetics, such as isoflurane, may contribute to ferroptosis through different mechanisms. Experimental evidence demonstrates that isoflurane exposure decreases transcription and protein expression of GPX4, a critical lipid repair enzyme in ferroptosis regulation. This down-regulation is accompanied by ROS generation, mitochondrial membrane potential disruption, and subsequent cell death [155].

These concentration and context dependent interactions highlight the need for further research to optimize anesthetic selection and dosing strategies for endothelial protection in perioperative settings. Additional studies are required to fully elucidate the clinical significance of anesthetic-ferroptosis interactions in vascular outcomes.

## 9. Conclusions

The literature review on the impact of anesthetics on endothelial function reveals complex mechanisms and interactions that can have both protective and detrimental effects. Anesthetics exhibit variable effects on the endothelium, depending on the dose and duration of administration. While some anesthetics demonstrate protective properties on the endothelium, particularly through increasing the synthesis of NO and antioxidant enzymes, others have the potential to induce endothelial dysfunction through pro-oxidative processes, immunomodulatory effects, and the stimulation of inflammation.

These findings are particularly significant in the perioperative period and in high-risk patient populations, where a certain degree of endothelial dysfunction is already present. Maintaining adequate endothelial function in such patients can play a crucial role in improving clinical outcomes. Therefore, understanding the mechanisms of action of different anesthetics on endothelial function is essential for optimizing perioperative care and improving treatment outcomes.

Future research should focus on elucidating the molecular mechanisms involved in the effects of anesthetics on endothelial function. Given the ongoing advancements in understanding these complex mechanisms, it is necessary to integrate new insights into clinical practice to enhance patient care quality. This approach allows for improved patient care quality, optimizing perioperative protection, and minimizing potential risks associated with anesthetic use.

## Data Availability

The data used to support the findings of this study are available from the corresponding author upon request.
